# Envenomation by *Trimeresurus stejnegeri stejnegeri*: clinical manifestations, treatment and associated factors for wound necrosis

**DOI:** 10.1590/1678-9199-JVATITD-2020-0043

**Published:** 2020-09-18

**Authors:** Liao-Chun Chiang, Wei-Jen Tsai, Po-Yu Liu, Cheng-Hsuan Ho, Hung-Yuan Su, Chih-Sheng Lai, Kuo-Lung Lai, Wen-Loung Lin, Chi-Hsin Lee, Yi-Yuan Yang, Uyen Vy Doan, Tri Maharani, Yan-Chiao Mao

**Affiliations:** 1 National Tsing Hua University, College of Life Sciences, Hsinchu, Taiwan.; 2 School of Medicine, National Defense Medical Center, Taipei, Taiwan.; 3 Division of Clinical Toxicology, Department of Emergency Medicine, Taichung Veterans General Hospital, Taichung, Taiwan.; 4 Division of Clinical Toxicology and Occupational Medicine, Department of Internal Medicine, Taipei Veterans General Hospital, Taipei, Taiwan.; 5 Institute of Environmental and Occupational Health Sciences, School of Medicine, National Yang-Ming University, Taipei, Taiwan.; 6 Division of Infection, Department of Internal Medicine, Taichung Veterans General Hospital, Taichung, Taiwan.; 7 Rong Hsing Research Center for Translational Medicine, National Chung Hsing University, Taichung, Taiwan.; 8 Department of Emergency Medicine, Tri-Service General Hospital, Taipei, Taiwan.; 9 Department of Emergency Medicine, E-Da Hospital, Kaohsiung, Taiwan.; 10 The School of Chinese Medicine for Post Baccalaureate, I-Shou University, Kaohsiung, Taiwan.; 11 Division of Plastic and Reconstructive Surgery, Department of Surgery, Taichung Veterans General Hospital, Taichung, Taiwan.; 12 Division of Allergy, Immunology and Rheumatology, Department of Internal Medicine, Taichung Veterans General Hospital, Taichung, Taiwan.; 13 Taichung Wildlife Conservation Group, Taichung, Taiwan.; 14 School of Medical Laboratory Science and Biotechnology, College of Medical Science and Technology, Taipei Medical University, Taipei, Taiwan.; 15 Graduate Program in Medical Biotechnology, College of Medical Science and Technology, Taipei Medical University, Taipei, Taiwan.; 16 Core Laboratory of Antibody Generation and Research, Taipei Medical University, Taipei, Taiwan.; 17 Department of Clinical Toxicology, Cho Ray Hospital, Ho Chi Minh City, Vietnam.; 18 Department of Emergency Medicine, Daha Husada Hospital, Kediri, East Java, Indonesia.

**Keywords:** Trimeresurus stejnegeri stejnegeri, Snakebites, Snake antivenom, Chinese green tree viper, Stejneger’s bamboo pitviper

## Abstract

**Background:**

*Trimeresurus stejnegeri stejnegeri* bite induces tissue swelling, pain, thrombocytopenia, rhabdomyolysis, and acute renal failure. However, the incidence of coagulopathy, factors associated with wound necrosis, and the appropriate management of this condition have not been well characterized yet.

**Materials:**

This study included patients bitten by *T*. *s*. *stejnegeri* that were admitted to the study hospitals from 2001 to 2016. Patient characteristics, laboratory data, and management approaches were compared in victims with and without wound necrosis.

**Results:**

A total of 185 patients were evaluated: three patients (1.6%) were asymptomatic; whereas tissue swelling and pain, local ecchymosis, wound necrosis, coagulopathy, thrombocytopenia, rhabdomyolysis, and renal impairment were present in 182, 53, 13, 15, 10, 1, and 3 patients, respectively. One patient died from coagulopathy and hemorrhagic shock. Antivenom was administered to all envenomed patients at a median time of 1.8 h after the bite. The median total dose of antivenom was five vials. Chi-square analysis showed that bitten fingers, using cold packs during first aid, presence of bullae or blisters, lymphangitis or lymphadenitis, local numbness and suspected infection to be significantly associated with wound necrosis. After adjustment using a multivariate logistic regression model, only cold packs as first aid, bulla or blister formation, and wound infection remained significant.

**Conclusions:**

The main effects of *T*. *s*. *stejnegeri* envenomation are tissue swelling, pain, and local ecchymosis. We do not recommend the use of cold packs during first aid to reduce wound pain, as this may be a risk factor for wound necrosis. In addition, patients with bulla or blister formation should be carefully examined for subsequent wound necrosis. Antiplatelet use may worsen systemic bleeding. No severe rhabdomyolysis or renal failure was observed in this large case series, we therefore considered that they were not prominent effects of *T*. *s*. *stejnegeri* bite.

## Background

Six venomous snakes are considered medically important in Taiwan: *Trimeresurus stejnegeri stejnegeri*, *Protobothrops mucrosquamatus*, and *Deinagkistrodon acutus* in the Crotalinae subfamily; *Daboia siamensis* in the Viperinae subfamily; and *Naja atra* and *Bungarus multicinctus multicinctus* in the Elapidae family [1]. More than 70% of the 1000 snake envenomations per year in this country are caused by the former two species [1,2]. *T*. *s*. *stejnegeri* is the only green pit viper in Taiwan, also found in India, Nepal, southern China, Vietnam and, possibly, Myanmar [3]. Its bite induces tissue swelling and pain, thrombocytopenia, rhabdomyolysis, and acute renal failure [4]. However, the severity of these symptoms has not been reported in previous studies [4]. Furthermore, the coagulopathy, high proportion (20%) of acute compartment syndrome and fasciotomy after bite [5], and the potential risk factors associated with wound necrosis have not been well established. The Taiwan government produces the only globally available antivenom for *T*. *s*. *stejnegeri* bites. As of 1999, the Taiwan Poison Control Center (PCC) suggests that 1 to 2 vials of specific antivenom should be administered to an envenomed patient. These guidelines, however, have not been scrutinized. In this study, we retrospectively analyzed data from 185 patients who were treated at two medical centers. The objective of this study was to facilitate a better understanding of the clinical manifestations and management approaches of *T*. *s*. *stejnegeri* bites among first-line clinicians.

## Materials

### Study population

Eligible patients were those admitted to Taichung Veterans General Hospital (VGH-TC) and Taipei Veterans General Hospital (VGH-TP) between January 2001 and September 2016. Possible cases of *T*. *s*. *stejnegeri* bite were identified by searching computer databases at VGH-TC and VGH-TP using the keyword “bamboo snake,” “*Viridovipera stejnegeri*” (previous name), and “*Trimeresurus stejnegeri*” in both English and Chinese, and these medical records carefully reviewed. Definite cases were classified by those diagnosed by examining the culprit snake, and suspected cases were classified by those diagnosed by having the patient identify the snake in a picture. Patients with snakebites for whom the culprit snake could not be identified, were excluded. Of note, some of the study population overlapped with a previous study reporting on a different time period [4].

The following data were extracted: sex, age, body part that was bitten, location at which the bite occurred, first aid used, clinical manifestation, laboratory finding, and management, including antivenom skin sensitivity test, adverse reaction to antivenom administration, time after bite to administration of antivenom, dosage of antivenom, indication for surgery, time after bite to first and last surgery, type of surgery, hospital stay, and follow-up period.

### Definition of variable

We extracted these variables from medical charts. If no anomaly was mentioned in the case notes, we assumed that none was present.

*Local sign or symptom*

The degree of swelling, measured using a modification of Blaylock’s classification [6], was categorized as minimal (local swelling at the bite site), mild (swelling involving the whole hand or foot), moderate (swelling involving the forearm up to the elbow or the leg up to the knee), or severe (swelling extending to the whole arm, thigh, or the area above). Acute compartment syndrome (ACS) was diagnosed when typical signs and symptoms and an intracompartmental pressure > 30 mmHg were documented [7]. Cases with only clinical signs and symptoms suggestive of ACS, without measurements of intracompartmental pressure, were classified as suspected ACS. Local numbness was described as effects that did not extend beyond the affected limb. Lymphangitis was clinically identified as a red line originating from the wound, whereas swollen tender lymph glands draining the affected area denoted lymphadenitis [8,9]. We used the same criteria for defining wound infection following snakebites as that used in previous studies [9,10], requiring at least two of the following three criteria: (1) onset of new or increasing pain, (2) localized erythema or swelling at the bite site (both of which were defined as an increase in severity if they occurred after the peak period and initial resolution of envenomation signs and symptoms), and (3) purulence at the bite site. The presence of fever (temperature ≥ 38°C, measured by tympanic thermometer) together with at least one of these three criteria also met the definition of a wound infection. If a patient underwent surgery, only pre-operative fever was included in the analysis. A wound discharge bacterial culture was performed when infection was suspected in a snakebite wound, and blood culture was performed during febrile episodes. The culture sampling technique has been described in the literature [11]. 

*Systemic signs or symptoms*

When the patients were admitted to hospital, they all underwent blood tests, including those to determine blood cell counts, prothrombin time (PT; international normalized ratio, INR), activated partial thromboplastin time (aPTT), creatine kinase (CK), and serum creatinine levels. These tests were performed repeatedly as required. If multiple test results were available, the most abnormal data were included in the analysis; if there were no abnormal data, data from the first test were included in the analysis. Coagulopathy was defined as INR > 1.4 (the upper limit of reference range in study hospitals) or aPTT > 35 s [12]. Thrombocytopenia was defined as platelet level < 150 × 10^9^/L [13]. D-Dimer elevation was defined as a value ≥ 0.55 fibrinogen equivalent unit (FEU) mg/L [12]. Hypofibrinogenemia was defined as fibrinogen antigen levels < 200 mg/dL (reference range: 200-400 mg/dL). Rhabdomyolysis was defined as CK levels > 1000 U/L, and renal impairment was defined as serum creatinine levels > 1.4 mg/dL [14]. 

*Outcome measurement*

Antivenom skin tests were performed according to manufacturer instructions. Early adverse reactions to antivenom, including anaphylaxis and urticaria, were defined as described in a previous study [15]. Late adverse reactions, such as serum sickness, were characterized by the presence of at least three of the following symptoms: fever, erythematous rash/urticaria, myalgia/arthralgia, headache, malaise, and nausea/vomiting 5-20 days post-antivenom treatment after carefully excluding infectious etiology [16].

### Statistical analysis

To identify the potential risk factors associated with wound necrosis, characteristic data of patients with and without wound necrosis were compared using the Mann-Whitney U test for continuous variables and the chi-squared test or Fisher’s exact test for categorical variables. Factors significantly associated with wound necrosis were then included in both univariate and multivariate logistic regression analyses and odds ratios (ORs), and relevant 95% confidence intervals (CIs) were reported. All data were analyzed using the Statistical Package for the Social Sciences, version 22.0 (2013 release; IBM Corp., Armonk, NY, USA). A two-tailed p-value < 0.05 denoted statistical significance.

## Results

### Characteristic data

One hundred and eighty-five cases of *T*. *s*. *stejnegeri* bites, including 125 from VGH-TC and 60 from VGH-TP, were examined. There were 20 definite and 165 suspected cases, 13 with wound necrosis, and 172 without wound necrosis (Table 1). In total, 124 patients (67%) were male. The median age was 53 years (range, 3-81). The bite occurred on a limb in all cases: 93 (50.3%) were bitten on their fingers and 15 (8.1%) on their toes. Of all patients, 33.5% were bitten in a field (e.g., bush, forest, and near river), 28.1% were bitten during farming activities, and only 2.2% were bitten while indoors. A few patients used methods of first aid, including: topical herbal remedies to treat the wound (5.9%); rope binding in an attempt to delay venom absorption (7.6%); incision and suction (3.2%); or cold packs to reduce pain (6.5%).

**Table 1.** Characteristic data, clinical manifestations, and laboratory findings of 185 *Trimeresurus stejnegeri stejnegeri* bite patients.

	Wound necrosis (n = 13)	No wound necrosis (n = 172)	Total cases (n = 185)	p-value*
Characteristic data				
• Male, n (%)	11	113	124 (67)	
• Age (years), median (range)	49 (36.5-63.5)	53.5 (3-81)	53 (3-81)	
Body part bitten, n (%)				
• Upper limb	13	133	146 (78.9)	
• Finger	11	82	93 (50.3)	0.01
• Lower limb	0	39	39 (21.1)	
• Toe	0	15	15 (8.1)	
Location, n (%)				
• Indoors	0	4	4 (2.2)	
• Yard/near home	1	10	11 (5.9)	
• Farm	4	48	52 (28.1)	
• Field (e.g. bush, forest, near river)	7	55	62 (33.5)	
• Other (e.g. temple, road)	1	55	56 (30.3)	
First aid, n (%)				
• Topical herbs	1	10	11 (5.9)	
• Rope binding^a^	2	12	14 (7.6)	
• Incision and suction	1	5	6 (3.2)	
• Cold packs	4	8	12 (6.5)	0.005
• Alcohol ingestion	0	2	2 (1.1)	
Clinical manifestations, n (%)				
• Asymptomatic	0	3	3 (1.6)	
Tissue swelling			182 (98.4)	
• Minimal	0	9	9 (4.9)	
• Mild	1	38	39 (21.1)	
• Moderate	8	75	83 (44.9)	
• Severe	4	47	51 (27.6)	
Acute compartment syndrome, suspected	0	2	2 (1.1)	
Local ecchymosis	5	48	53 (28.6)	
Bullae/blister	8	14	22 (11.9)	< 0.001
Lymphangitis/lymphadenitis	4	17	21 (11.4)	0.045
Local numbness	4	16	20 (10.8)	0.038
Fever (≥ 38°C)	1	9	10 (5.4)	
Wound infection	9	12	21 (11.4)	< 0.001
• Any positive bacterial culture	5 / 7^d^	1 / 5	6 / 12	
Laboratory findings				
• Coagulopathy, n (%)	0	15	15 (8.1)	
• INR, median (range)^b^; n	NA^e^	3.4 (1.9-6.2); n = 8	8 (4.3)	
• aPTT(s), median (range)^b^; n	NA	38 (35.7-47.7); n = 10	10 (5.4)	
• Thrombocytopenia (× 10^9^/L), median (range), n (%)	NA	128 (76-148); n = 10	10 (5.4)	
• D-Dimer elevation, n	4 / 4	21 / 23	25 / 27	
• Blood level (mg FEU/L), median (range)^c^	1.1 (0.7-5.1)	1.3 (1.1-2.5)	1.1 (0.7-5.1)	
• Hypofibrinogenemia	1 / 4	9 / 28	10 / 32	
• Blood level (mg/dL), median (range)	126	109 (33-182)	117.5 (33-182)	
Rhabdomyolysis	0	1	1 (0.5)	
Renal dysfunction	0	3	3 (1.6)	

*Only values of statistical significance are shown. ^a^Including any form of rope, rubber band, or towel/clothes bindings. ^b^One patient with INR > 10, and another two patients with aPTT > 224 s were excluded for calculation. ^c^One case with D-Dimer > 10 FEU mg/L was excluded for calculation. ^d^Bacterial culture was obtained in seven patients and five had positive results. ^e^NA: not applicable because there was no case.

### Local effect

Three patients (1.6%) were asymptomatic. Local swelling and pain were observed in all other patients. Local ecchymosis at the bite site, bullae or blisters, lymphadenitis or lymphangitis, local numbness, fever, and suspected wound infection occurred in 28.6%, 11.9%, 11.4%, 10.8%, 5.4%, and 11.4% of patients, respectively. Positive bacterial cultures were noted in 6/12 patients from whom cultures were obtained. Seven organisms were identified (Table 2): *Staphylococcus aureus*, *Staphylococcus hominis*, *Citrobacter freundii*, *Enterobacter cloacae*, *Morganella morganii*, *Pseudomonas aeruginosa*, and an anaerobic *Veillonella* sp. Each were identified in a single patient except for *E*. *cloacae* which was identified in two. Polymicrobial infection by *S*. *aureus*, *E*. *cloacae*, and *Veillonella* sp. was observed in one patient. Antibiotics administered before obtaining cultures included cefazolin, ceftriaxone, oxacillin, amoxicillin/clavulanic acid, clindamycin, and metronidazole.

**Table 2.** Surgical outcome or wound bacteriology in eight envenomed patients.

Case number	Body part bitten	Clinical manifestations	Intravenous antibiotic administered before surgery	Type of surgery	Time after bite to surgery in days	Hospital stay in days	Outcome	Bacterial culture
Case 1	Finger	Swelling and pain, ecchymosis, lymphadenitis/lymphangitis, bullae/blister, fever, wound necrosis, wound infection	Cefazolin and metronidazole	Debridement < 5 cm	7	11.9	Recovery	Wound swab and blood culture: both negative
Case 2	Finger	Swelling and pain, wound necrosis, wound infection	Ceftriaxone and clindamycin	Debridement < 5 cm	7	10	Recovery	Wound swab after debridement: *Pseudomonas aeruginosa*
Case 3	Finger	Swelling and pain, ecchymosis, bullae/blister, wound necrosis, wound infection	Amoxicillin/clavulanic acid	Debridement 5-10 cm	11	7.6	Recovery	Wound swab after debridement: *Enterobacter cloacae*
Case 4	Finger	Swelling and pain, bullae/blister, wound necrosis, wound infection	Amoxicillin/clavulanic acid	Debridement < 5 cm Debridement < 5 cm Debridement < 5 cm Full-thickness skin graft	3 13 21 24	5.9	Recovery	Wound swab after first debridement: *Morganella morganii*
Case 5	Finger	Swelling and pain, ecchymosis, bullae/blister, local numbness, wound necrosis, wound infection	Oxacillin	Debridement < 5 cm	6	8.8	Recovery	Bullae fluid: *Staphyloccocus hominis*
Case 6	Forearm	Swelling and pain, ecchymosis, local numbness, suspected ACS^a^	Cefazolin and gentamicin	Fasciotomy Debridement > 10 cm and split-thickness skin graft Revision of scar	0.33 5 59	11.8	Recovery	Not performed
Case 7	Finger	Swelling and pain, wound infection	No antibiotic	Not performed	-	8.1	Recovery	Joint fluid: *Citrobacter freundii*
Case 8	Finger	Swelling and pain, local numbness, lymphadenitis/lymphangitis, bullae/blister, wound necrosis, wound infection	Cefazolin and metronidazole	Not performed	-	3	Recovery	Wound swab: *Staphylococcus aureus*, *Enterobacter cloacae*; *Veillonella* sp. (anaerobic)

a Acute compartment syndrome.

### Systemic effect

Coagulopathy was present in 15 patients (8.1%); serial blood coagulation tests were performed in 8 of the 15 patients, and all 8 patients recovered within 1-8 days. Fresh frozen plasma (FFP) was administered in addition to the antivenom to two of these patients (one with INR > 10 and aPTT 28.1 s and the other with INR = 5.5 and aPTT > 224 s), with favorable outcomes. One patient died due to coagulopathy (INR = 6.2 and aPTT > 224 s) and hemorrhagic shock, did not received FFP transfusion in time. Thrombocytopenia occurred in 10 patients (5.4%), including 9 classified as mild and 1 moderate decrease in platelet levels. D-Dimer elevation and hypofibrinogenemia was present in 24/26 and 10/32 patients tested, respectively. Rhabdomyolysis and renal impairment occurred in 1 (0.5%, highest CK level, 1679 U/L) and 3 (1.6%, serum creatinine 1.7, 2, and 3.1 mg/dL) patients, respectively. During the study period, no hemolysis, delayed systemic bleeding, or gastrointestinal effect were observed.

### Management and outcome

Of the 182 envenomed patients, three patients (1.6%) with a negative antivenom skin test developed anaphylaxis during antivenom administration presenting as generalized urticaria in all three, stridor in two, and hypotension in one, which promptly resolved upon epinephrine, antihistamine, steroid, and fluid replacement therapy (Table 3). Seven patients (3.8%) had skin rashes without systemic reactions, however only two had a positive skin test result. The positive and negative likelihood ratios of the antivenom skin test for anaphylaxis and skin rashes were 0 and 1.1 (CI 95% 1.1**-**1.2), and 3 (CI 95% 0.9**-**10.5) and 0.8 (CI 95% 0.5**-**1.3), respectively. Ten patients experienced serum sickness after receiving a median of 10 vials of antivenom (interquartile range, IQR, 4.5-12.5). This median dosage of antivenom administration differed significantly in patients without serum sickness (median 4 vials, IQR 2-8; Mann-Whitney U test, p-value 0.041). One hundred sixty-nine patients (92.9%) received specific antivenom within 6 h after the bite. The exact time of first antivenom administration could be extracted in 111 envenomed cases, and the median time elapsing between the bite and administration of antivenom was 1.8 h (IQR, 1.2-3.3 h). The median total dose of specific antivenom was five vials (IQR, 2**-**8 vials).

Surgical indications included wound necrosis with secondary infection in five patients and suspected ACS in the other one. A total of 11 surgeries were performed in these six cases, including six debridement, one fasciotomy, and two split-thickness skin graft (STSG) or full-thickness skin graft (FTSG), alongside scar revision. No finger or toe amputation was observed. Excluding the suspected ACS case, the first surgery was performed a median of 7 days after bite (IQR, 4.5**-**9 days), and the last was performed 24 days after bite. Two patients (1.1%) had suspected ACS in the forearm: one underwent fasciotomy at 8 h post-bite, debridement and STSG on day 5, and scar revision on day 59 (hospital stay, 11.8 days), whereas the other refused surgery but recovered without complications. The median hospital stay was 2.4 days (IQR, 1.4**-**4 days) for all patients, including 2.2 days (IQR, 1.3**-**3.8 days) for unoperated patients and 9.4 days (IQR, 7.2**-**11.8 days) for operated patients. The median follow-up period was 8.5 days (IQR, 5**-**14.3 days). No case of tetanus was observed in the study.

**Table 3.** Management of 182 *Trimeresurus stejnegeri stejnegeri* envenomed patients.

	Wound necrosis (n = 13)	No wound necrosis (n = 169)	Total cases (n = 182)	p-value
**Specific antivenom administration**				
Antivenom skin test, n (%)				
• Negative	13	146	159 (87.4)	
• Positive	0	18	18 (9.9)	
• Not performed	0	5	5 (2.7)	
Allergy to antivenom, n (%)				
• Anaphylaxis	1	2	3 (1.6)	
• Skin rash	0	7	7 (3.8)	
• Serum sickness	1	9	10 (5.5)	
Time after bite to first antivenom dose, (h)				0.137
• < 6 h (n, %)	11	158	169 (92.9)	
• 6-12 h	0	6	6 (3.3)	
• > 12 h	2	5	7 (3.8)	
• Median in h, IQR	2.3 (0.8-28.4)	1.8 (1.3-3.3)	1.8 (1.2-3.3)	0.952
Total antivenom dose in vials, median (IQR)	4 (2.5-9)	5 (2-8)	5 (2-8)	0.996
Operation case, n (%)	5	1 (fasciotomy)	6 (3.3)	< 0.001
• Time after bite to first surgery, median (IQR)	7 (4.5-9)	0.33		
• Time after bite to last surgery, median (IQR)	24; n = 1^b^	59		
• Debridement	5	1	6 (3.3)	< 0.001
• Fasciotomy	0	1	1 (0.5)	
• STSG/FTSG^a^	1	1	2 (1.1)	
• Scar revision	0	1	1 (0.5)	
Hospital stay in days, median (IQR)	5.9 (3.1-9.4)	2.2 (1.3-3.8)	2.4 (1.4-4)	0.002
• Unoperated case	3.1 (2.1-6)	2.2 (1.3-3.8)	2.2 (1.3-3.8)	
• Operated case	8.8 (6.8-11)	11.8	9.4 (7.2-11.8)	
Outpatient follow-up case, n (%)	5	71	76 (41.8)	0.802
• Follow-up in days, median (IQR)	13.5 (7.8-16.8)	8 (5-12)	8.5 (5-14.3)	0.088

a Split-thickness skin graft and full-thickness skin graft. ^b^A single case received four operations on days 3, 13, 21 (debridement), and 24 (FTSG) post-bite.

### Statistical findings

To better identify the factors associated with wound necrosis, we included only envenomed cases in the regression analysis. Univariate logistic regression showed that finger as the bite site, using cold packs, bullae or blister, lymphangitis or lymphadenitis, local numbness, and wound infection were significant (Table 4). Multivariate logistic regression using a forward stepwise selection model approach showed that only cold packs, bullae or blister, and wound infection were significant associated with wound necrosis. In the envenomed cases, early administration of antivenom (< 6 h) was not shown to be associated with a lower incidence of bullae or blister formation, wound necrosis, coagulopathy, and thrombocytopenia (p-value = 0.055, 0.235, 0.306, and 1, respectively) using chi-squared analysis.

**Table 4.** Logistic regression analysis of associated factors for wound necrosis in 182 envenomed patients.

Variable	Wound Necrosis			
	Crude OR	p-value	Adjusted OR	p-value
Finger as the bite site	6.1 (1.3-28.4)	0.021	-	-
Cold packs	8.9 (2.3-35.6)	0.002	26.5 (3.4-205.6)	0.002
Bullae or blister formation	17.7 (5.1-61.5)	< 0.001	9.1 (1.8-46.7)	0.008
Lymphangitis or lymphadenitis	4.0 (1.1-14.3)	0.035	-	-
Local numbness	4.3 (1.2-15.4)	0.027	-	-
Wound infection	29.3 (7.8-109.1)	< 0.001	23.7 (4.1-135.7)	< 0.001

- Not significant.

## Discussion

The hallmarks of *T*. *s*. *stejnegeri* bites are tissue swelling and pain, probably resulting from histamine release and microvascular leakage triggered by phospholipase A_2_s (PLA_2_s) and other enzymes [17]. Tissue swelling can be so severe that it imitates ACS, leading to treatment by fasciotomy. The reported incidence of ACS and fasciotomy following snakebite in recent Taiwanese studies ranges from 6.6% to 39% [18,19], including 20% following *T*. *s*. *stejnegeri* bites [5], unlike the figures of 0.2% to 1.36% reported in worldwide studies [18] and < 1.1% in the present study. The method of diagnosing ACS was not mentioned in the study by Hsieh et al [5]. Diagnosis based on signs and symptoms is unreliable, as snake venom can induce limb edema, pain, and/or paresthesia, which confound the diagnostic accuracy of physical examination [18,19]. One of our two suspected ACS patients did not receive fasciotomy but recovered well with repeated antivenom therapy, and the other who received fasciotomy without repeated antivenom administration, needed revision of his scar to restore his hand function. To avoid unnecessary surgery like that reported in Taiwan, we recommend monitoring intracompartmental pressure in snakebite victims with suspected ACS [8,20]. Surgery should be considered when the intracompartment pressure was documented to be > 30 mmHg or other objective criteria have been met [7,20]. Antivenom may reduce intracompartmental pressure; therefore, an adequate dose of antivenom should be administered prior to attempting decompression surgery [8,21].

Wound necrosis following *T*. *s*. *stejnegeri* bite probably results from the cytotoxic effect of venom PLA_2_s and/or secondary infection [22]. Either a local deposit of the venom which would be inaccessible to the antivenom, or a mechanism which is no longer under the direct influence of the venom like NETosis and/or oxidative stress related to the pathological amplification of the inflammatory syndrome in the continuation, even the late appearance of tissue destruction should also be considered [23,24], as this may render the antivenom therapy to be ineffective. Nevertheless, the contributing effect of cold packs has not been thoroughly examined [25]. In Taiwan, cold packing was occasionally used during first aid by snakebite patients to reduce pain [9]. Although ‘cryotherapy’ (soaking the whole limb in ice or ice water), is prohibited in snakebite management [26]. Placing cold packs intermittently on the bite site for pain control, was believed to be less likely to cause harm [27]. Nonetheless, the optimal temperature, frequency, and anatomic location of cold pack application has not been evaluated in snakebite management as is has for jellyfish stings [28]. Such first aid may impair tissue perfusion, causing wound necrosis, and should be discouraged until sufficient evidence is available. Bullae and blister formation was associated with wound necrosis is similar to the finding of a previous study [29], which may result from the proteolytic effect of snake venom metalloproteinases (SVMPs) at the dermal-epidermal junction [30]. From a clinical perspective, bullae or blister formation usually precedes wound necrosis in *T*. *s*. *stejnegeri* bite. Early removal of the bullae or blister, similar to the management of a chemical burn, may reduce the venom load at the inoculation site [31,32]. However, whether such a procedure lowers the risk of wound necrosis should be evaluated.

Chen et al. studied the bacteriology of snakebite wounds in Taiwan and found that *Morganella morganii* and *Enterococcus* spp. were the most commonly isolated pathogens. As such, they suggested amoxicillin/clavulanic acid with ciprofloxacin or piperacillin/tazobactam to treat snakebite wound infection [33]. Mao et al. further specifically investigated the bacteriology of *N*. *atra* bite wounds, and found that these two pathogens were primarily related to *N*. *atra* bite infection [9,10]. In our study, *M*. *morganii* and *Enterococcus* spp. were not commonly observed. These differences in wound bacteriology may be due to interspecies variation in the oral flora of the related snakes [10]. A higher infection rate was observed in our study compared to a previous study (11.4% vs 6%) [4], however we defined wound infection based on the criteria used by Mao et al. [9,10], and it is noteworthy that 10%**-**70% of inanimate object puncture wounds develop infectious complications [34]. Based on the bacteriology findings, we suggest a first-generation cephalosporine with aminoglycoside, or sulfamethoxazole/trimethoprim as initial antibiotics for the management of infected *T*. *s*. *stejnegeri* bite wounds. Prophylactic antibiotics administration is not recommended and antibiotics should be administered only when infection occurs.

*T*. *s*. *stejnegeri* venom consists of SVMPs (43.1%), PLA_2_s (24.5%), snake venom serine proteases (SVSPs, 11%), and other minor components [35]. The median lethal dose of crude venom is 2 μg/g intraperitoneal injection in mice [36]. SVMPs cause proteolytic degradation of extracellular matrix which normally maintains the structure and integrity of capillaries. This degradation leads to the disruption of microvessel networks, edema, and hemorrhage [35]. SVSPs are hemostatically active toxins perturbing the maintenance and regulation of both the blood coagulation cascade and the fibrinolytic feedback system at specific points [37]. PLA_2_s have evolved into extremely potent toxins, displaying myotoxic, anticoagulant, and edema-inducing activities [17,38]. In *T*. *s*. *stejnegeri* venom, thrombin-like enzymes (TLEs) and α- and β-fibrinogenases are present [39]. TLEs are pseudo-procoagulant SVSPs [40]; in animal studies, when TLEs are administered intravenously, they induce rapid defibrinogenation via proteolytic effects on fibrinogen, forming fibrin monomers (non-cross-linked fibrin) that can be rapidly removed from the circulation either by fibrinolysis or the reticuloendothelial system, thus prolonging the coagulation time [41-43]. Alpha- and β-fibrinogenases, which are SVMPs and SVSPs, respectively, cause fibrinogenolysis and fibrinolysis, and so potentiate the effect of TLEs on net anticoagulation [39,44]. Prothrombin activation inhibitor, an acidic PLA_2_, interferes with prothrombin and its activating factors via reversible binding [45]. PLA_2_s and SVSPs may synergistically work to potentiate the hemorrhagic activity of SVMPs [46].

Although ecchymosis has been reported in *T*. *s*. *stejnegeri* bites [4], systemic bleeding is rare. In the present study we observed that ecchymosis usually occurred around the wound, probably because the coagulotoxin levels (e.g., TLEs, fibrinogenases, and plasminogen activator) are low [35,41,47], despite the high proportion of hemorrhagic SVMPs in the venom [35]. Despite this, an unfortunate incident still occurred. A 71-year-old woman who was receiving aspirin therapy for coronary artery disease sustained a *T*. *s*. *stejnegeri* bite on her left foot. After the bite, she had an accidental fall resulting in facial contusion, and she visited the hospital 13 h after envenomation. Physical examination on arrival revealed local ecchymosis around the wound in addition to leg swelling. She had clear consciousness and brain computed tomography uncovered no intracranial hemorrhage. However, coagulopathy and upper gastrointestinal bleeding occurred, therefore, five vials of antivenom were immediately administered. She died 18 h after hospitalization as a result of continuous gastrointestinal bleeding and shock. Her initial blood fibrinogen level and coagulation factor 2, 7, 9, and 10 activities were 32.5 mg/dL, 40% (reference range, 50-150%), 48.1% (65-135%), 48.2% (60-140%), and 27.7% (45-155%), respectively. Platelet count was normal. We speculated that *T*. *s*. *stejnegeri* venom influences the extrinsic coagulation pathway in addition to causing hypofibrinogenemia. In our observation, thrombocytopenia was uncommon and generally mild. The mechanism might have involved platelet aggregation/agglutination and the action of SVMPs on the microvessel wall [48,49]. Antiplatelet therapy may worsen severe bleeding [50], while administration of FFP in patients with *T*. *s*. *stejnegeri* bite and coagulopathy may help to restore coagulation factors and reduce the risk of bleeding [8,51].

Three patients experienced renal impairment, including the 71-year-old woman who died from hemorrhagic shock. Her renal impairment (serum creatinine level up to 3.1 mg/dL) was assumed to have resulted from hypovolemia. One 80-year-old male patient experienced transient renal impairment (serum creatinine levels up to 1.7 mg/dL), and his serum creatinine returned to normal within 4 days after fluid replacement therapy, suggesting that the impairment was related to dehydration. Another 80-year-old male patient who had creatinine levels of 2 mg/dL, was later found to have chronic renal insufficiency through a kidney sonography. Despite the presence of myotoxic and hemolytic PLA_2_s in *T*. *s*. *stejnegeri* venom [22,48], no significant rhabdomyolysis, or hemolysis was observed. Although acute renal failure has been reported in *T*. *s*. *stejnegeri* bites, the suspected cause was not described [4,5]. After careful review of the medical records, we believe that significant rhabdomyolysis and severe nephrotoxicity are not typical features of *T*. *s*. *stejnegeri* envenomation.

The Taiwan government produces four types of antivenom, all of which are ammonium sulfate-precipitated F(ab’)_2_ fragment in lyophilized form: two bivalent specific antivenoms (one for *P*. *mucrosquamatus* and *T*. *s*. *stejnegeri* and one for *N*. *atra* and *B*. *m*. *multicinctus*), and two monovalent antivenoms (one for *D*. *acutus* and one for *D*. *siamensis*). The bivalent specific antivenom for *T*. *s*. *stejnegeri* and *P*. *mucrosquamatus* was determined to neutralize 26 mg venom of *T*. *s*. *stejnegeri* per vial [36]. Based on the range amount of venom milked from a snake (0.6-30.3 mg), Taiwan PCC has stated that 1**-**2 vials are required to treat an envenomed bite [36]. Our antivenom dosage was higher than that recommended by PCC, probably because of the higher proportion of moderate and severe swelling and finger or toe bites in our study than that in a previous study [52]. Additionally, physicians may believe that the use of antivenom accelerates recovery [53]. However, the lack of an association between early administration of antivenom and a lower incidence of certain pathological effects (i.e., bullae or blister formation, wound necrosis, coagulopathy, and thrombocytopenia) remains poorly understood. 

Our findings should be evaluated in a properly designed prospective study. Additional tests of the antivenom against the enzymes related to cytotoxicity, the effect of venom on individual coagulation factors, and the ability of antivenom to neutralize the coagulotoxic effect specifically and determine variations in the venom components could be the initial steps to optimizing the treatment for *T*. *s*. *stejnegeri* bites. Although a greater proportion of patients in our study had an allergic response to antivenom in comparison with that reported during management of *N*. *atra* bite [9], there was a low incidence of anaphylaxis. In addition, neither hospital had adopted a desensitization protocol or administration of prophylactic epinephrine because of the low incidence of anaphylaxis. Our findings suggest that antivenom skin tests do not accurately predict allergic responses and may be omitted. Instead, close observation of all patients who receive antivenom is warranted [8]. 

### Limitations

This study had limitations. First, VGH-TC, and VGH-TP are medical centers. The incidence of symptomatology as well as the management of cases may not be generalizable to all primary care facilities due to possible referral bias. Secondly, coagulation factor activities were not routinely measured and because the blood tests were usually repeated as requested by clinicians rather than at fixed time intervals, the onset, and duration of certain hematological disturbances may not have been identified. Thirdly, patients may have received several forms of treatment (e.g., wound cleansing, or topical herbal remedies) that may have altered the bacterial composition and load before bacterial culture collection. Furthermore, because the data collection was from retrospective chart review and the absence of data was recorded as the occurrence of signs or symptoms not being present, the results should be cautiously interpreted. Despite these limitations, it is the first study to specifically investigate *T*. *s*. *stejnegeri* bites, with findings that may have important clinical implications for the management of this condition.

## Conclusions

The main effects of *T*. *s*. *stejnegeri* envenomations are tissue swelling, pain, and local ecchymosis. The factors associated with wound necrosis were cold packing as first aid, bulla and blister formation, and wound infection. With treatment using specific antivenom, life-threatening bleeding is rare, and rhabdomyolysis and nephrotoxicity are not prominent consequences of *T*. *s*. *stejnegeri* envenomation.

### Abbreviations

ACS: acute compartment syndrome; aPTT: activated partial thromboplastin time; *B. m. multicinctus*: *Bungarus multicinctus multicinctus*; CI: confidence interval; CK: creatine kinase; *D*. *acutus*: *Deinagkistrodon acutus*; *D*. *siamensis*: *Daboia siamensis*; *E*. *cloacae*: *Enterobacter cloacae*; FEU: fibrinogen equivalent unit; FTSG: full-thickness skin graft; INR: international normalized ratio; IQR: interquartile range; *N*. *atra*: *Naja atra*; OR: odds ratio; PCC: poison control center; PLA_2_: phospholipase A_2_; *P*. *mucrosquamatus*: *Protobothrops mucrosquamatus*; PT: prothrombin time; *S*. *aureus*: *Staphylococcus aureus*; STSG: split-thickness skin graft; SVMP: snake venom metalloproteinase; SVSP: snake venom serine protease; TLE: thrombin-like enzyme; *T*. *s*. *stejnegeri*: *Trimeresurus stejnegeri stejnegeri*; VGH-TC: Taichung Veterans General Hospital; VGH-TP: Taipei Veterans General Hospital.
